# The impact of Independent Component Analysis on TMS-evoked potentials: a within-subject comparison across motor and prefrontal areas

**DOI:** 10.1016/j.cnp.2026.03.007

**Published:** 2026-04-04

**Authors:** Eva Oostra, Emile d’Angremont, Timo van Hattem, Shilpa Anand, Sophie Schubert, Odile A. van den Heuvel, Ysbrand D. van der Werf

**Affiliations:** a Amsterdam UMC, Vrije Universiteit Amsterdam, Dept. Psychiatry, De Boelelaan 1117, Amsterdam, the Netherlands; b Amsterdam UMC, Vrije Universiteit Amsterdam, Dept Anatomy & Neuroscience, De Boelelaan 1117, Amsterdam, the Netherlands; c GGZ inGeest Specialized Mental Health Care, Amsterdam, the Netherlands; d Amsterdam Neuroscience, Mood, Anxiety, Psychosis, Sleep & Stress program, Amsterdam, the Netherlands; e Hertie-Institute for Clinical Brain Research, University of Tübingen, Germany; f Department of Neurology & Stroke, University of Tübingen, Germany; g Child and Adolescent Psychiatry and Psychosocial Care, Emma Children’s Hospital, Amsterdam UMC, Amsterdam, the Netherlands; h Brain Research and Innovation Centre, Ministry of Defence, the Netherlands; i Department of Psychiatry, University Medical Centre Utrecht Brain Centre, the Netherlands; j Amsterdam Neuroscience, Compulsivity Impulsivity Attention program, Amsterdam, the Netherlands

**Keywords:** Transcranial magnetic stimulation, Electroencephalography, Prefrontal cortex, Motor cortex, Healthy subjects

## Abstract

**Background:**

Independent component analysis (ICA) is a common method to remove artifacts and improve signal quality in transcranial magnetic stimulation combined with electroencephalography (TMS-EEG) data. However, the impact of applying different rounds of ICA for TMS-EEG datasets are lacking. Here, we investigated the impact of applying zero, one, or two rounds of ICA on TMS-evoked potentials (TEPs) in the motor cortex and prefrontal cortex using a within-subject design.

**Methods:**

Twenty-three healthy participants received 51 single-pulses over the left primary motor cortex (M1) and dorsolateral prefrontal cortex (DLPFC), with simultaneous EEG-recording. Data were preprocessed three times: with zero, one, or two rounds of ICA. We compared the TEP-amplitudes and local mean field potential-area under the curve (LMFP-AUC).

**Results:**

After M1 stimulation, preprocessing two ICA rounds resulted in significantly more N40-peaks identified, and smaller P30 (3.84 µV ± 0.78) and P60 (3.08 µV ± 0.96) amplitudes compared to 0 ICA rounds. After DLPFC stimulation, zero ICA rounds led to significantly fewer identified P30 (n = 4) and N40-peaks (n = 5), and larger P60 (5.63 µV ± 1.34) and P180-amplitudes (8.62 µV ± 0.76) compared to 0 ICA. The N100-component remained stable across ICA conditions for both brain areas.

**Discussion:**

Our results showed a large impact of ICA, compared to zero ICA rounds, particularly after DLPFC stimulation; likely due to increased eye-blink artifacts. We therefore recommend implementing eye-blink artefact removal during the first round of ICA. In M1, the addition of an ICA round only impacted the early-TEPs. This study is the first to directly compare ICA-effects on both motor and non-motor TMS-EEG data using a within-subject design.

## Highlights

• First within-subjects study on impact of independent component analysis (ICA)
• Different rounds of ICA have distinct effects on TMS-evoked potentials in DLPFC & M1.
• Eye-blink removal in the first ICA round improves DLPFC TEPs.

## 1 Introduction

Since its introduction in the previous century, transcranial magnetic stimulation combined with electroencephalography (TMS-EEG) has become a widely used tool to investigate the brain’s electrophysiologic responses to TMS (Belardinelli et al., 2021). A number of instrumental adaptations have been made since then (Bonato et al., 2006, Thut et al., 2005, Virtanen et al., 1999), followed by optimization of preprocessing steps to eliminate TMS pulse-related artifacts while preserving the TMS evoked potential (TEP) as much as possible (Rogasch et al., 2017). TMS-EEG has primarily been applied to the primary hand-motor area (M1), where supra-threshold stimulation elicits a contralateral motor evoked potential (MEP) measurable via electromyography (EMG) (Farzan and Bortoletto, 2022). A single TMS pulse administered to M1 elicits a TEP response lasting ∼ 300 ms; comprising positive and negative deflections, i.e. P30, N40, P60, N100 and P180 peaks. TMS-EEG has also been applied in clinical research settings; serving as a tool to identify biomarkers for treatment response or underlying pathophysiological properties of the disorder (Cao et al., 2021). In particular, stimulation of the dorsolateral prefrontal cortex (DLPFC) has shown promise; for instance, the baseline N100 amplitude in the left DLPFC predicts remission of suicidal ideation after magnetic seizure treatment in depression (Sun et al., 2016).

In order to optimally benefit from the application of TMS-EEG in clinical and research practice, there is a need for international consensus on both data acquisition and processing. Considering data processing, different pipelines have emerged in order to systematically eliminate artifacts from the dataset (see ref. (Hernandez-Pavon et al., 2023) and (Bertazzoli et al., 2021) for an overview). The majority of these pipelines make use of independent component analysis (ICA). ICA is used to detect independent time-locked events and assess whether the event is contaminated with artifacts or noise (Bertazzoli et al., 2021). These can then be filtered out, to be left with a more ‘true’ cortical signal (Farzan and Bortoletto, 2022). Generally, the first ICA round is aimed to identify and reject large TMS-evoked muscle/electrical artifacts. The second round is aimed to filter out electrode noise, eye movement, eye-blinks, smaller muscle artifacts and reflections of other than brain physiology (e.g. cardiovascular) (Rogasch et al., 2017). Bertazzoli et al. (2021) investigated whether TMS-EEG outcome would differ when it was processed with different pipelines. They found differences in suppression of the artifacts, differences in sensitivity of the TEP peak detection, and reproducibility of the TEPs. The original TESA pipeline (Rogasch et al., 2017), that uses two ICA rounds, together with the SOUND-SSP-SIR pipeline (Mutanen et al., 2018) showed better results compared to the other pipelines. More recently, Beck et al. (Beck et al., 2024) provided a comprehensive overview of methodological choices and their effect on TEPs after single pulse TMS (spTMS) over M1. Analyzing 124 studies with a total of 1892 participants (median n = 12 per study), they found that the number of ICA rounds significantly affected TEP amplitudes, particularly the N100 peak. However, the need for within-subject replication remains. In this study, we build on those findings by investigating the impact of zero, one or two ICA rounds – using the TESA pipeline (Rogasch et al., 2017) – on TEPs, without changing any other protocol parameter. spTMS was administered on the left M1 and DLPFC in healthy adult participants. As TMS-EEG outcome measures we used 1) the number of peaks identified after the different ICA rounds, 2) the amplitudes and latencies of the individual TEPs, and 3) the local mean field power (LMFP). We hypothesize to see more artifact-related measures (e.g. larger amplitudes of the TEPs) when we do not apply any ICA rounds.

## 2 Methods

### 2.1 Study population

A group of 33 participants was screened for this study and were included when there was no contra-indication for MRI and TMS application, no psychiatric diagnoses according to the SCID-5 and no psychotropic medication was taken at least for the past 12 months (see Supplementary section 1.1 for details on the inclusion criteria). All participants provided informed consent. The study adhered to the principles outlined in the Declaration of Helsinki and was in accordance with the Medical Research Involving Human Subjects Act (in Dutch: WMO).

### 2.2 Study design

This study was conducted on three different days. The informed consent procedure and screening questionnaires were performed on day 1. On the second day, the participants underwent cognitive assessments before entering the MRI, as well as two cognitive tasks (i.e., the Tower of London task (Heuvel et al., 2005) and the Stop Signal task (de Wit et al., 2012)) during the functional MRI scan. The peak activation in the left DLPFC during the Tower of London task was used for the determination of the stimulation location at the TMS-EEG session. On the third day, participants underwent the TMS-EEG session. See Supplementary section 1.1 for details about the MRI and TMS-EEG acquisition and the cognitive assessments.

#### 2.2.1 TMS-EEG acquisition

TMS pulses were delivered using a MagStim BiStim^2^ stimulator along with a figure-of-eight D70 alpha coil (The Magstim Co.Ltd., Whitland, Wales). This article focuses on to left DLPFC and M1 stimulation with spTMS; for the complete study design, see Supplementary section 1.5.

The resting motor threshold (rMT) was determined using EMG from the right first dorsal interosseous muscle. M1 stimulation was targeted at the motor hotspot, and the rMT was defined as the minimum intensity eliciting ≥ 50 µV MEPs in at least 50% of trials. Left DLPFC stimulation was guided by neuronavigation (Localite TMS Navigator GmbH, Bonn, Germany), using task-based fMRI coordinates (see Supplementary section 1.2 and 1.3).

Each region received 153 pulses: 51 single TMS pulses (1 ms, at 120% MT, monophasic waveform), and 102 double pulse paradigm pulses (see Supplementary section 1.4). This was applied over the left M1, left DLPFC, left preSMA and right DLPFC (the latter two not discussed in this article), with randomized inter-trial-intervals between 3–5 s. The order of brain areas was randomized per participant. EEG was recorded using a 64-channel EasyCap system (international 10–20 layout), with FCz as reference, and AFz as ground. Data were sampled at 10 kHz using a Neuvo 64-channel amplifier (Compumedica Neuroscan, Germany), with electrode impedances kept below 5 kΩ. The long axis of the coil was placed in a 45-degree angle from the midline during stimulation.

**Fig. 1:**
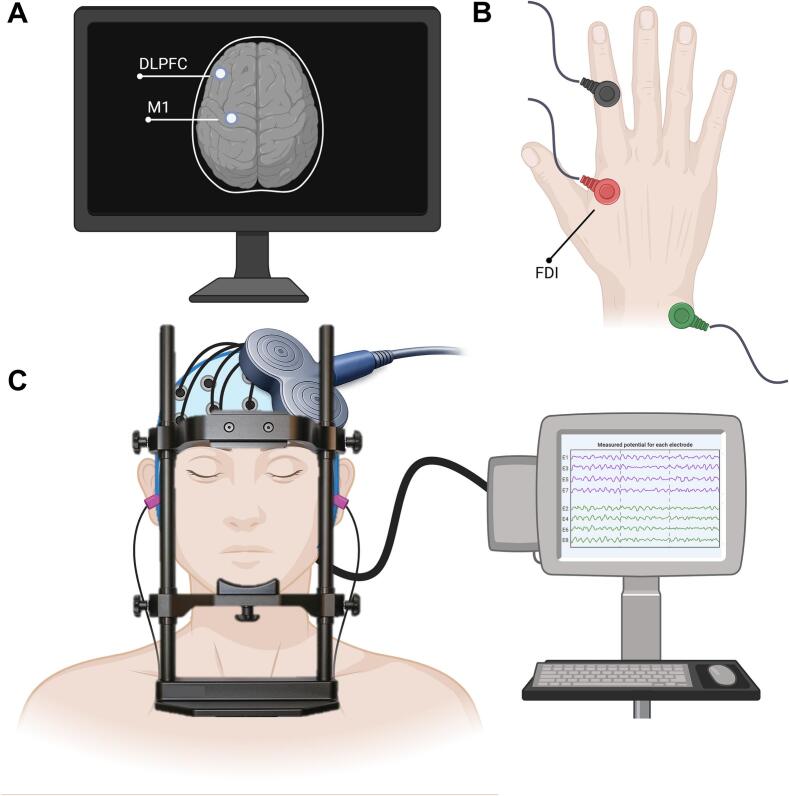
**Experimental set-up of a participant during the TMS-EEG measurement.** (A) During the TMS-EEG measurement, neuronavigation is used to localize the brain areas of interest (left M1 and DLPFC). (B) Parallel to M1 stimulation with TMS-EEG, EMG is used to measure the corresponding MEPs. On the right hand, the red electrode is placed on the first dorsal interosseous (FDI), the black electrode is placed on the proximal interphalangeal joint of the index finger, and the green electrode on the pisiform bone. (C) During stimulation, the participant’s head is resting on a chin-rest, with a band around the head to give support. The eyes are closed, and active noise canceling through white noise is played via earbuds. Created in https://BioRender.com. TMS = transcranial *magnetic stimulation, EEG = electroencephalography, M1 = primary motor cortex, DLPFC = dorsolateral prefrontal cortex, EMG = electromyography, MEP = motor evoked potential.* (For interpretation of the references to colour in this figure legend, the reader is referred to the web version of this article.)

During the TMS-EEG session, participants were seated in front of a small table on which they could rest their arms. To minimize head movement artifacts on the EEG signal, the participants’ head was placed in a chin bar with forehead rest and supported using a strap attached to the chin bar (see Fig. 1). To minimize auditory evoked potentials in the EEG data, active noise masking was applied by playing white noise through MRI eligible earplugs. Participants were asked to close their eyes during the stimulation.

### 2.3 Preprocessing of acquired TMS-EEG data

The preprocessing steps followed the original TESA toolbox steps – as described by Rogasch et al. (2017) – in EEGLAB running in Matlab 2022b. See Table 1 for more details of the different steps and our scripts on [https://github.com/emiledangremont/TMS-EEG]. The electrodes for our region of interests (ROIs) included F3, F1, FC3 and FC1 for the left DLPFC (Avnit et al., 2023, Chung et al., 2017, Hoy et al., 2021) and C1 and C3 for M1 (Silva et al., 2021). The time windows were determined to account for a relatively large heterogeneity in latencies of the different peaks, but not to overlap with windows of neighboring peaks of the same polarity. They were determined as follows: for P30 [20–40 ms], N40 [30–55 ms], P60 [45–75 ms], N100 [85–145 ms] and P180 [160–220 ms].

**Table 1:** Analysis steps according to TESA toolbox (Rogash et al., 2017).

1. Import data		
2. Automated removal of bad electrodes	*Based on (1) a flat line for longer than 5 s, (2) noise with a frequency of more than 4 times standard deviation, (3) correlation with nearby channels of less than 0.8 and (4) kurtosis value of more than 4 times standard deviation.*	
3. Epoch data	*From −1500 till 1500 ms*	
4. Baseline correction	*Use −800 till −110 ms*	
5. Find TMS pulse	*Check whether stimulation was administered at each event*	
6. Remove TMS pulse artifact and peaks of TMS-evoked muscle activity	*From −5 till 15 ms*	
7. Interpolate missing data around TMS pulse	*Cubic interpolation fitted on 1 ms before and after missing data*	
8. Downsample data	*1 kHz*	
9a. Automated removal of bad trials	*Based on joint probability with a threshold of 3 times standard deviation*	
9b. Visual removal of bad trials		
10. Replace interpolated data around TMS pulse with constant amplitude data		
**11. ICA round 1: remove large amplitude artifacts**	***TMS-evoked muscle, electrical, and movement artifacts (including eye blinks)***	
12. Interpolate missing data around TMS pulse after ICA 1	*Cubic interpolation fitted on 10 ms before and after missing data*	
13. Band-pass and band-stop filter data	*Band-pass: 1*–*80 Hz* *Band-stop: 48*–*52 Hz*	
14. Visual removal of bad trials		
15. Replace interpolated data around TMS pulse with constant amplitude data		
**16. ICA round 2: remove all other artifacts**	***Smaller muscle activity, eye movements, electrode noise, non-brain derived reflections of physiology (*e.g. *a heartbeat)***	
17. Interpolate missing data around TMS pulse after ICA 2	*Cubic interpolation fitted on 10 ms before and after missing data*	
18. Interpolate missing channels	*Spherical interpolation*	
19. Re-reference to average	*From FCz as reference, to re-referencing to the average of all electrodes*	
20. Baseline correction	*Use −110 till −15 ms*	
21. Extract ROI	*Left DLPFC: F1, F3, FC1, FC3* *Left M1: C1, C3*	
22. Find peaks	*P30: 20*–*40 ms* *P60: 45*–*75 ms* *P180: 160*–*220 ms*	*N40: 30*–*55 ms* *N100: 85*–*145 ms*
23. Output results	*Amplitude and latency of peaks*	
24. Plot the results		

*TMS = transcranial magnetic stimulation, ICA = independent component analysis,*

*M1 = primary motor cortex, DLPFC = dorsolateral prefrontal cortex*.

#### 2.3.1 ICA steps

For the aim of our study, we repeated the preprocessing of the TMS-EEG dataset three times; one with two rounds of ICA (including step 11, 12, 15 and 16 in Table 1), one with one round of ICA (including step 11,12, excluding step 15 and 16, Table 1) and one with zero rounds of ICA (excluding step 11, 12, 15 and 16, Table 1). During the first round of ICA (step 11, Table 1), large TMS-evoked muscle and electrical artifacts were identified and rejected. We noticed that DLPFC stimulation caused more stimulation-related artifacts, which could be due to facial muscle contraction or blink-related eye movements – which can occur even when the eye is already closed (Collewijn et al., 1985). We coined these artifacts 'blink artifacts' and decided to reject them already in the first round of ICA, which is contrary to the TESA toolbox (Rogasch et al., 2017) (see examples in Supplementary section 1 Fig. S1 and S2). The second round of ICA was used for residual artefacts caused by muscle activity, eye movements, electrode noise and non-brain derived reflections of physiology (e.g. heartbeat). Visual inspection of the identified components was performed by four different raters (EO, EdA, WS and MS) who were intensively trained and regularly discussed unclear cases in order to reach consensus.

### 2.4 Outcome measures and statistical analysis

For the analysis, data were averaged over trials and over electrodes within the ROI. The following TMS-EEG outcome measures were extracted per subject, per brain area, per ICA application: identification, latency and amplitude of *a priori* determined TEP peaks, and the area under the curve of the local mean field power (LMFP-AUC). Peaks were defined as a datapoint larger or smaller than +/- five data points within the predefined time-window. If multiple peaks were found, the largest was selected. For our main analysis, we investigated whether the individual TEPs could be identified after zero, one or two rounds of ICA, per brain area, using Cochrane’s Q test with McNemar’s test post hoc. Secondly, we analyzed the impact of 0, 1 or 2 ICA rounds, on TEP amplitude, latency and LMFP-AUC. For both left M1 and DLPFC, LMFP-AUC was calculated for early LMFP (25 ms-80 ms after the TMS pulse), and late LMFP (81 ms-270 ms after the TMS pulse). Differences in outcome measures across ICA conditions were tested using repeated measures ANOVA, with post hoc comparisons performed with paired-T test when p < 0.05. P-values were corrected for multiple testing via FDR correction.

### 2.5 Sensitivity analysis

Using the butterfly plot (which consists of all subject-level averaged TEP-responses, see Fig. S6/S7) we visually checked for outliers. If any outliers were detected (and therefore excluded from the primary analyses), a sensitivity analysis was done including the outlier.

## 3 Results

### 3.1 Demographics of sample

Of the 33 participants that were screened, two were not eligible based on SCID diagnosis, and two had a contra-indication for the MRI. Three of the 29 inclusions withdrew their consent after the MRI scan was performed due to personal circumstances and one before the MRI scan was performed, leaving a sample of 25 healthy participants that completed both the MRI and TMS-EEG visits. Unfortunately, the first two datasets were corrupted and therefore unusable; the final dataset consisted of 23 participants (mean age 37.00 (±13.66) years and 56.6% women). One outlier in TEP responses of one participant following M1 stimulation was identified (see Fig. S3). No outlier was found after DLPFC stimulation. We decided to exclude this participant's data from the main analysis for M1 but retained the DLPFC data. One participant did not complete the left DLPFC stimulation, resulting in 22 datasets for both M1 and DLPFC stimulation. For sensitivity analysis, please refer to the supplementary materials, where we show the complete M1 dataset. The mean percentage of epochs used in the analyses after M1 stimulation was 90.2% (±9.3%) without applying ICA, 90.5% (±8.5%) after 1 round of ICA and 91.9% (±4.1%) after two rounds of ICA. After DLPFC stimulation, the mean number of epochs used in the analyses was 93.2% (±4.4%) without applying ICA, 91.1% (±7.3%) after 1 round of ICA and 88.7% (±7.5%) after two rounds of ICA.

### 3.2 Impact of zero, one and two rounds of ICA on grand mean

**Fig. 2:**
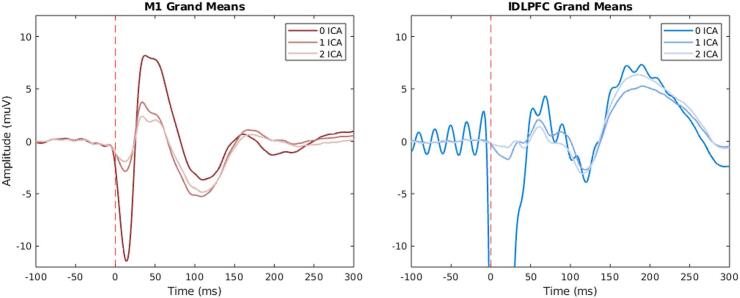
**Grand means after different ICA applications.** On the left the grand mean after left M1 stimulation in red after 0 ICA rounds (dark contrast), 1 ICA round (medium contrast) and 2 ICA rounds (light contrast). On the right the grand mean of left DLPFC stimulation in blue after 0 ICA rounds (dark contrast), 1 ICA round (medium contrast) and 2 ICA rounds (light contrast). *ICA = independent component analysis, M1 = primary motor cortex, lDLPFC = left dorsolateral prefrontal cortex.* (For interpretation of the references to colour in this figure legend, the reader is referred to the web version of this article.)

As shown in Fig. 2, different rounds of ICAs applied to the dataset have different impacts on the grand mean. By visual inspection, one can see that the grand mean after 0 ICA rounds for both M1 and DLPFC exhibits larger peaks at the early phase of the TEP response, especially for the DLPFC, where the first peak has a very large amplitude (±-400 µV). The grand mean after DLPFC stimulation without ICA, shows a large ringing artifact around the first peak, which appeared in the data after the band-stop filter between 48–52 Hz (see Fig. S5). See Fig. S6/S7 for the butterfly plots.

#### 3.2.1 Detection of individual peaks in TEP signal

We investigated whether the individual TEPs were identified for every participant (n = 22 for both brain areas). By visual inspection, one can see that the largest impact on the number of identified peaks is seen for the early peaks after both M1 and DLPFC stimulation. P-values are corrected by FDR.

##### 3.2.1.1 Differences in number of peaks identified

After M1 stimulation, the Cochrane’s Q test was significant for the P30 (Q = 6, p = 0.049) and N40 peak (Q = 10.67, p = 0.0048). Post hoc, we found a trend level difference in number of P30 peaks identified after 0 rounds of ICA (n = 15) versus 1 ICA round (n = 20, p_uncorrected = 0.073). This did not survive the FDR correction (p = 0.22). For the N40 peaks, we found a statistical significant difference between the number of peaks identified after 0 rounds of ICA (n = 9) compared after 2 ICA rounds (n = 17, p = 0.040).

**Fig. 3:**
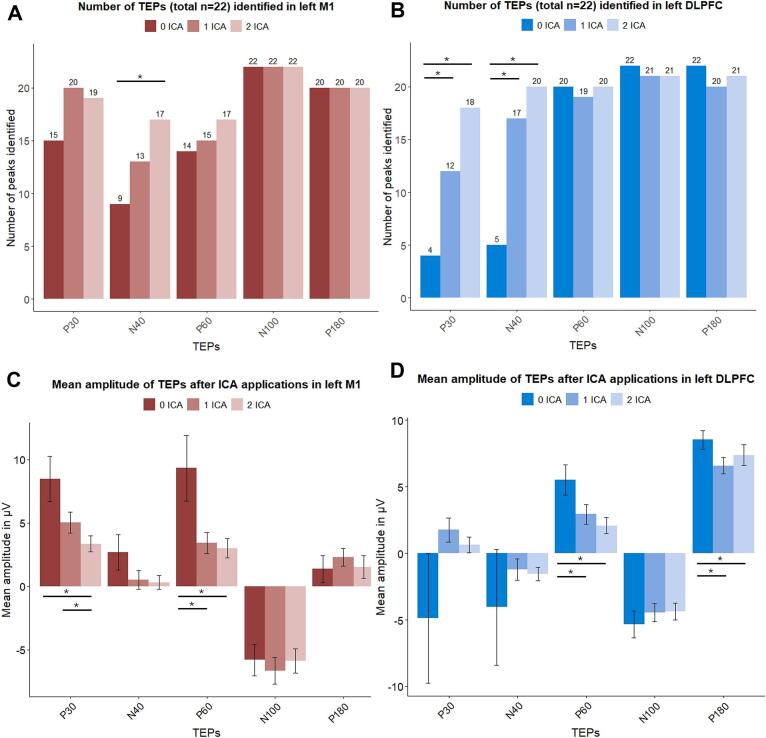
**Bar plots with number of TEPs identified (A&B), and mean amplitudes of the identified TEPs (C&D), across different ICA applications after M1 and DLPFC stimulation.** A and B: number of individual TEPs identified after 0 ICA rounds (dark contrast), 1 ICA round (medium contrast) and 2 ICA rounds (light contrast) after M1 (red) and DLPFC stimulation (blue). C and D: Mean amplitude of the TEPs identified after 0 ICA rounds (darker contrast), 1 ICA round (medium contrast) and 2 ICA rounds (light contrast) after M1 (red) and DLPFC (blue) stimulation. Data is shown in mean ± standard error of the mean. Differences were calculated using Cochrane’s Q test (A&B) or repeated measures ANOVA test (C&D). Significance was reached when p < 0.05 with FDR correction. *TEPs = TMS evoked potentials, ICA = independent component analysis, M1 = primary motor cortex, lDLPFC = left dorsolateral prefrontal cortex, ANOVA = analysis of variances.* (For interpretation of the references to colour in this figure legend, the reader is referred to the web version of this article.)

After DLPFC stimulation, the Cochrane’s Q test was significant for the P30 (Q = 16.44, p = 0.00027) and N40 peak (Q = 22.24, p < 0.0001). Post hoc, we found that the number of identified P30 and N40 peaks after 0 rounds of ICA (four peaks and five peaks, respectively) was statistically significantly lower than after 1 ICA round (P30: 12 peaks, p = 0.040 and N40: 17 peaks, p = 0.005) and after 2 ICA rounds (P30: 18 peaks, p = 0.003 and N40: 20 peaks, p = 0.0009). See Fig. 3A and B.

#### 3.2.2 TEP latencies and amplitudes

Secondly, we investigated the impact of different ICA rounds on amplitudes and latencies of individual TEPs after M1 and DLPFC stimulation. See Fig. 3C&D and Fig. S3 for respectively mean amplitudes after 0, 1 or 2 ICA rounds, and mean latencies after 0, 1 or 2 ICA rounds for the individual TEPs. Results are shown in mean ± standard error.

##### 3.2.2.1 ANOVA analyses

The repeated measures ANOVA showed statistically significant differences in peak amplitudes between different ICA rounds for P30 (F = 9.60, p = 0.0008), N40 (F = 4.49, p = 0.028), and P60 (F = 5.28, p = 0.01) following M1 stimulation, and for P60 (F = 6.17, p = 0.005) and P180 (F = 6.11, p = 0.005) following DLPFC stimulation. After M1 stimulation, we found post hoc a statistically significantly lower amplitude of the P30 peak after 2 ICA rounds (3.84 µV ± 0.78) compared to 1 ICA round (6.36 µV ± 0.96, p = 0.0006) and 0 ICA rounds (8.89 µV ± 1.86, p = 0.0006). Furthermore, the amplitude of the N40 peak was trend-wise larger (2.68 µV ± 1.40) after 0 rounds of ICA compared to 1 round of ICA (0.51 µV ± 0.73, p_uncorrected = 0.076), or 2 rounds of ICA (0.32 µV ± 0.56, p_uncorrected = 0.05). Similar results were found for the P60 peak after 0 ICA rounds (10.20 µV ± 2.92) compared to 1 ICA round (3.07 µV ± 0.89, p = 0.048) or 2 ICA rounds (3.08 µV ± 0.96, p = 0.048). P-values from the N40 amplitudes did not survive FDR correction.

After DLPFC stimulation, we found post hoc a statistically significantly larger amplitude of the P60 after 0 ICA rounds (5.63 µV ± 1.34) compared to 1 ICA round (2.96 µV ± 0.83, p = 0.034) or 2 ICA rounds (2.14 µV ± 0.70, p = 0.025). Also, the P180 peak showed a larger amplitude after 0 rounds of ICA (8.62 µV ± 0.76) compared to 1 ICA round (6.59 µV ± 0.61, p = 0.023) or 2 ICA rounds (7.42 µV ± 0.81, p = 0.025). No statistically significant differences were found for the latencies of the TEPs between the different ICA applications (see Table 2).

**Table 2:** **Results of the amplitudes and latencies of the individual TEPs after applying 0, 1 or 2 ICA rounds.** Differences were compared using repeated measures ANOVA and post hoc paired-*t* test with FDR correction. Statistical significance was reached when p ≤ 0.05.

**M1**		Amplitude (µV)					Latency (ms)		
TEPs		0 ICA *M (SE)*	1 ICA *M (SE)*	2 ICA *M (SE)*	F (p)	Post Hoc	0 ICA *M (SE)*	1 ICA *M (SE)*	2 ICA *M (SE)*
	P30	8.89 (1.86)	6.36 (0.96)	3.84 (0.78)	**9.60** **(<.001)**	**2 ICA < 0 ICA** **2 ICA < 1 ICA**	32.8 (1.17)	32.6 (1.06)	32.7 (1.02)
	N40	2.68 (1.40)	0.16 (0.65)	−0.10 (0.41)	**4.49 (0.028)**	none	42.1 (2.12)	42.7 (2.37)	42.0 (2.22)
	P60	10.20 (2.92)	3.07 (0.89)	3.08 (0.96)	**5.78 (0.01)**	**0 ICA > 1 ICA** **0 ICA > 2 ICA**	54.6 (2.64)	55.5 (2.17)	55.6 (2.43)
	N100	−5.80 (1.23)	−6.66 (1.05)	−5.89 (0.96)			113.2 (3.43)	109.8 (3.32)	110.4 (3.39)
	P180	1.63 (1.09)	2.25 (0.76)	1.53 (0.99)			182.5 (3.70)	181.4 (3.81)	187.6 (3.85)

**DLPFC**		Amplitude (µV)					Latency (ms)		

TEPs		0 ICA *M (SE)*	1 ICA *M (SE)*	2 ICA *M (SE)*	F (p)	Post Hoc	0 ICA *M (SE)*	1 ICA *M (SE)*	2 ICA *M (SE)*
	P30	−7.57 (11.0)	2.50 (1.90)	3.25 (2.34)			33.5 (0.5)	31.5 (2.5)	31.0 (2.0)
	N40	−3.48 (5.57)	−1.06 (2.25)	−0.86 (2.16)			44.0 (3.34)	43.25 (2.21)	43.5 (2.22)
	P60	5.63 (1.34)	2.96 (0.83)	2.14 (0.70)	**6.17 (0.005)**	**0 ICA > 1 ICA** **0 ICA > 2 ICA**	59.6 (1.93)	57.24 (1.42)	56.71 (1.71)
	N100	−5.50 (1.12)	−4.53 (0.72)	−4.46 (0.67)			117.1 (3.22)	118.3 (2.97)	114.8 (2.75)
	P180	8.62 (0.76)	6.59 (0.61)	7.42 (0.81)	**6.11 (0.005)**	**0 ICA > 1 ICA** **0 ICA > 2 ICA**	182.9 (3.22)	187.3 (3.64)	187.2 (3.48)

*TEPs = TMS evoked potentials, ICA = independent component analysis, M1 = primary motor cortex, lDLPFC = left dorsolateral prefrontal cortex, M = mean, SE = standard error, ANOVA = analysis of variances, FDR = False discovery rate*.

#### 3.2.3 LMFP-AUC

We investigated the impact of 0, 1 or 2 ICA rounds on the magnitude of the LMFP-AUC. For both stimulation locations, we found that the early and late LMFP-AUC after 0 ICA rounds was statistically significantly larger compared to the LMFP-AUC after 1 or 2 ICA rounds. No statistically significant differences were found between the LMFP-AUC after 1 and 2 ICA rounds, except for the early LMFP-AUC after DLPFC stimulation, see Table 3.

**Table 3:** **Results of the LMFP-AUC magnitude after applying 0, 1 or 2 ICA rounds.** Differences were compared using repeated measures ANOVA and post hoc paired-*t* test with FDR correction. Statistical significance was reached when p ≤ 0.05.

**M1**	Amplitude						Statistics		
LMFP-AUC	0 ICA *M (SE)*		1 ICA *M (SE)*		2 ICA *M (SE)*		F (p)		Post Hoc
Early	417 (72.8)		197 (24.0)		153 (22.1)		**10.77 (< 0.001)**		**0 ICA > 1 ICA** **0 ICA > 2 ICA**
Late	851 (111.0)		708 (96.1)		668 (100.0)		**7.38 (0.002)**		**0 ICA > 1 ICA** **0 ICA > 2 ICA**
					

**DLPFC**									

LMFP-AUC		0 ICA *M (SE)*		1 ICA *M (SE)*		2 ICA *M (SE)*		F (p)	Post Hoc
Early	601 (84.5)		191 (20.0)		140 (14.6)		**27.37 (< 0.001)**		**0 ICA > 1 ICA > 2 ICA**
Late	1253 (93.8)		899 (70.7)		884 (82.3)		**15.79 (< 0.001)**		**0 ICA > 1 ICA** **0 ICA > 2 ICA**

*TEPs = TMS evoked potentials, ICA = independent component analysis, M1 = primary motor cortex, lDLPFC = left dorsolateral prefrontal cortex, M = mean, SE = standard error, ANOVA = analysis of variances, FDR = false discovery rate*.

### 3.3 Sensitivity analysis

One TEP response after M1 stimulation (from one participant) showed a huge artifact, as well as a ringing artefact without applying ICA rounds. This was absent in the TEP responses of the other participants after M1 stimulation, which is why it was considered an outlier. As seen in Fig. S4, the outlier stands out in the butterfly plot and severely impacts the grand mean.

To overcome the ringing effect, which is prominently present after DLPFC stimulation, we explored the effect of applying a bandpass filter of 1–48 Hz and no bandstop filter. See Fig. S5 for the butterfly plots and grand means.

## 4 Discussion

With this study, we aimed to assess the impact of various rounds of ICA on TEPs using a within-subject design, with spTMS administered on the left M1 and left DLPFC. To our knowledge, this is the first to investigate this in both a motor and a non-motor (prefrontal) brain area using this design. We found that TEP outcomes are differently affected by the number of ICAs applied. This is particularly apparent for early peaks. The largest impact was seen between applying any and not applying ICA rounds. Small differences were found between applying one ICA round and two ICA rounds.

### 4.1 Impact of ICA on TMS-EEG outcome variables

The number of ICA rounds differentially affected TMS-EEG outcome measures depending on whether M1 or DLPFC was stimulated. Without applying ICA, TEPs after M1 stimulation showed a trend towards fewer identifiable N40 peaks and larger amplitudes of the P30, N40, and P60 components, compared to applying one or two ICA rounds. This is in line with the findings of Beck et al. (Beck et al., 2024). After DLPFC stimulation, no application of ICA resulted in fewer identifiable P30 and N40 peaks, and larger amplitudes of the P60 and P180 components, compared to applying one or two ICA rounds. The larger amplitudes of early peaks likely result from residual TMS-related artifacts and eye blinks that were present in the data in the absence of ICA, consistent with the review by Beck et al. (Beck et al., 2024). The use (and non-use) of ICA did not affect the latencies of the individual TEPs, across brain areas.

The effect of applying one versus two rounds of ICA was largely comparable, as it only significantly influenced the amplitude of the P30 component after M1 stimulation. The difference in P30 amplitude was not reflected in the LMFP-AUC analyses; the early LMFP-AUC magnitude did not differ significantly between one or two rounds of ICA. This indicates that the impact of one versus two rounds of ICA on this marker of general local cortical excitability is minimal, but may still influence individual TEP components. In contrast, after DLPFC stimulation, we observed a statistically significant difference in early LMFP-AUC magnitude between one and two ICA rounds, while the individual amplitudes of the corresponding TEPs (P30, N40, P60), showed no statistically significant differences. It may be possible that there is a differential impact on the individual early TEP amplitudes after DLPFC stimulation between applications of ICA, but that the statistical comparison is underpowered due to the reduced number of identified peaks. The differential impact of 1 vs 2 ICA rounds between brain regions could be explained by the number of eyeblink artifacts that are rejected after DLPFC stimulation vs. M1 stimulation. As shown in the Supplementary Fig. 1, there is a large difference in percentage variance of the suspected eyeblink artifact between DLPFC stimulation and M1 stimulation. This results in more rejected components, and therefore more EEG signal, during the first round of ICA after DLPFC stimulation, which potentially influences the amplitude of the early peaks.

N100 measures remained stable across all ICA rounds and stimulation sites, which contrasts with Beck et al. (Beck et al., 2024), who reported a significant effect of ICA on N100 amplitudes. Their dataset, however, included studies with varying protocols, including different stimulation intensities (subthreshold, on threshold, suprathreshold) and noise masking applications (none, passive, active). They found that these differences reflected alterations in amplitude of the N100 peak and the N100-P180 complex. In our study, the different processing protocols were applied to one dataset, using active noise masking and stimulation at 120% MT. Our findings therefore suggest that, under these conditions, N100 amplitude is robust to artifact contamination and ICA processing, after both M1 and DLPFC stimulation.

### 4.2 Considerations for processing

Several anticipated and unanticipated challenges were encountered during data processing. When no ICA rounds were applied, substantial TMS-related artifacts were observed, including motor drifts and ringing artifacts (Rogasch et al., 2017). The latter were especially pronounced following DLPFC stimulation, and in one case after M1 stimulation; this case was considered an outlier and excluded from the M1 dataset. Ringing artifacts typically occur when a band-stop filter (in our case, 48–52 Hz) is applied to data containing high-amplitude deflections (Rogasch et al., 2017). These were effectively prevented after applying at least one round of ICA (before filtering). Independent of brain region, it is important to remove high-amplitude deflections caused by electrode noise or muscle artifact in TMS-EEG data. This will not only prevent a ringing artifact when applying a band-stop filter, but also prevent overestimation or misinterpretation of early TEPs.

It is important to note that our considerations apply specifically to the application of the original TESA pipeline for processing TMS-EEG data. TESA is one of the handful TMS-EEG processing pipelines that are publicly available (Bertazzoli et al., 2021, Hernandez-Pavon et al., 2023), and like the majority it applies two rounds of ICA. However, there is an ongoing debate about the conceptual validity of applying ICA during TMS-EEG processing. ICA relies on the assumption that signal sources are statistically independent, e.g. that neural activity is independent from artifactual signal. This assumption is problematic; TEPs and most of the artifacts are time-locked to the administered TMS pulse, which violates this assumption of independence (Hernandez-Pavon et al., 2023). The SOUND-SSP-SIR pipeline (Mutanen et al., 2018) largely replaced the ICA rounds by the SOUND and SSP-SIR algorithms that uses *a priori* models of neural activity and from electrophysiological noise, to selectively suppress artifacts without decomposing the data into components that require subjective interpretation. Unlike ICA, this pipeline does not rely on the assumption of independence and are less susceptible to inter-rater variability (Bertazzoli et al., 2021, Hernandez-Pavon et al., 2023). This algorithm is now also applicable in the TESA pipeline (Mutanen et al., 2020). However, each method comes with trade-offs; SOUND-SSP-SIR pipeline tends to remove signal suspected to contain artifacts more rigorously, increasing the risks of removing data that contains neural activity. The TESA pipeline retains more signal, with the risk of preserving more residual artifacts. When comparing the outputs of these pipelines, the differences were profoundly present in the amplitudes and waveforms of early TEP components (Bertazzoli et al., 2021). These differences likely reflect the varying degrees and strategies of artifact removal, mainly affecting the early peaks. This mirrors the differences we observed in our results. The optimal pipeline for TMS-EEG processing may strongly depend on the stimulation protocol and acquisition parameters.

### 4.3 Limitations and strengths

To the best of our knowledge, this study is the first to assess the impact of differential rounds of ICA on TMS-EEG outcome measures after M1 and DLPFC stimulation using a within-subject design. This allows us to solely study the impact of ICA, without other protocol parameters being different (e.g. stimulation intensity, noise masking, etc.). Using this design, we were not able to investigate the impact of different noise masking applications or stimulation intensities, and other methodological choices – among others discussed by Beck et al. (2024). With 51 spTMS pulses per brain region, our study has administered relatively fewer pulses (e.g. the 124 studies reviewed by Beck and colleagues showed a median of 100 pulses). This methodological decision was driven by the duration of the full stimulation protocol, which consisted of 153 pulses in total (covering spTMS and two ppTMS paradigms), applied across four different brain regions (see Supplementary). Each region took ∼ 25 min to complete. Although a higher number of pulses potentially yield more robustness and a higher signal to noise ratio (Noda et al., 2024), we demonstrated that delivering 51 spTMS pulses per participant were sufficient to generate a typical TEP response. Still, data quality varied across participants, resulting sometimes in small amount of usable data. We chose not to exclude these participants to maintain statistical power.

To limit operator bias in component selection, all raters were trained in component recognition and rejection, and questionable components were discussed among raters. Nonetheless, some inter-rater variability is likely to be unavoidable.

Finally, although we aimed to position the TMS coil at a 45 degree angle to the sagittal midline, this was not always feasible at the left DLPFC site due to interference from the chin rest. While the 45 degree angle is not considered the optimal angle for DLPFC stimulation (Cerins et al., 2024), different angles may increase or reduce artifacts (Parmigiani et al., 2025).

### 4.4 Recommendations for future studies

Our results demonstrate that ICA is necessary to discard large TMS-evoked electrical and muscle artifacts, especially when no other method for removing high-amplitude deflections is applied. We found small differences between 1 and 2 rounds of ICA. Nonetheless, results may significantly change when adding a second round of ICA, especially for early TEPs. Therefore, deciding on the number of ICA rounds in processing TMS-EEG data should be done carefully, and by taking the quality of the data into account. Prefrontal brain stimulation resulted in more artifacts, leading to larger differences throughout the whole TEP response compared to M1 stimulation. Therefore, we suggest including eye-blink removal during the first round of ICA, especially after prefrontal stimulation.

Although our sample size was sufficient to obtain TEP responses, larger replication studies are needed to compare methodological decisions and work towards standardized preprocessing methodology.

## 5 Declaration of Generative AI and AI-assisted technologies in the writing process

During the preparation of this work the author(s) used ChatGPT in order to improve the readability and language of the manuscript (grammar checks and shortening of sentences). Additionally, we used ChatGPT to improve the TMS-coil icon (mainly its angle) for Fig. 1. After using this tool/service, the author(s) reviewed and edited the content as needed and take(s) full responsibility for the content of the published article.

## 6 CRediT Authorship contribution statement

**Eva Oostra:** Conceptualization, Data curation, Formal analysis, Investigation, Methodology, Validation, Project administration, Resources, Visualization, Writing – original draft, Writing – review & editing. **Emile d’Angremont:** Conceptualization, Data curation, Formal analysis, Software, Investigation, Methodology, Validation, Project administration, Resources, Visualization, Writing – original draft, Writing – review & editing. **Timo van Hattem:** Methodology, Software, Investigation, Writing – review & editing. **Shilpa Anand:** Methodology, Software, Investigation, Writing – review & editing. **Sophie Schubert:** Methodology, Software, Investigation, Writing – review & editing. **Odile A. van den Heuvel:** Conceptualization, Funding acquisition, Writing – review & editing, Supervision, Validation, Project administration, Resources. **Ysbrand D. van der Werf:** Conceptualization, Formal analysis, Project administration, Resources, Writing – review & editing, Validation, Supervision.

## Declaration of competing interest

The authors declare that they have no known competing financial interests or personal relationships that could have appeared to influence the work reported in this paper.
