# Supplementary material for: The impact of Independent Component Analysis on TMS-evoked potentials: a within-subject comparison across motor and prefrontal areas
Source: Clin Neurophysiol Pract. 2026 Apr 4;11:273–81. doi: 10.1016/j.cnp.2026.03.007 (PMC13091408; doi:10.1016/j.cnp.2026.03.007)
Supplement: Supplementary Data 1 [file mmc1.docx]

Supplementary Materials

**The impact of Independent Component Analysis on TMS-EEG outcomes: A Within-Subject comparison across Motor and Prefrontal Stimulation**

Oostra & d’Angremont *et al.*

*corresponding author* [*e.oostra@amsterdamumc.nl*](mailto:e.oostra@amsterdamumc.nl)

1. Methods

- 1. *Participants*

*In- and exclusion criteria*

Participants were included when 1) age was between 18-65 years old and 2) no psychiatric, neurodevelopmental or neurological diagnosis was present (assessed using the Structured Clinical Interview for the Diagnostic and Statistical Manual of Mental Disorders IV Axis I disorders (SCID)), or if there was a personal history of DSM-5 diagnosis, except for a diagnosis of depression or anxiety longer than 12 months ago; (3) use of psychotropic medication within the last 12 months; (4) contraindications to MRI such as pregnancy, metal in the teeth, iron in the body and claustrophobia, (5) contraindications to TMS, e.g. epilepsy or family history of epilepsy (first-degree family member), metallic implanted devices, any neurological disorder that causes a lesion in the brain, head trauma resulting in unconsciousness for at least one hour, and previous brain surgery, pregnancy or breastfeeding, syncope, use of pro-convulsive medication or medication affecting the cortical excitability, sleep deprivation, severe heart disease, comorbid substance use/dependence/abuse, resting motor threshold higher than 75% of maximum stimulator output, or no useful motor evoked potential (MEP) eligible.

*1.1 Cognitive assessment battery*

The following tasks were conducted before entering the MRI scanner: the Dutch translation of the National Reading Test (to estimate ones IQ), Visual spatial N-back task (to assess working memory), Emotional Stroop task (to measure selective attention and processing speed), and Temporal Discounting and Risk Choice task (to assess risk preferences). After the cognitive assessment battery, participants practiced the computerized Tower of London (TOL) and Stop-signal task (SST) as preparation for the executing during the MRI scan.

*Tower of London task*

The TOL paradigm used in this study was the same as described in Fitzsimmons et al., (Fitzsimmons et al., 2025). The fMRI computerized task consisted of five different planning conditions, varying in difficulty, and one counting condition as the control condition. During the planning conditions the participants were required to plan a sequence of moves to match the beads of the begin situation to the goal situation. The five planning conditions differed in amount of moves needed to reach the goal image; from level 1 (one move needed) till level 5 (five moves needed). During the counting condition, the participant had to count the number of yellow and blue beads presented in the image. The different conditions were presented in a pseudorandomized order, ensuring that each planning condition with level 3 or higher was followed by a counting condition. The stimulus presentation was self-paced, with a maximum response time of 60 seconds; the total task length was fifteen minutes.

*Stop-signal task*

The SST paradigm consisted of 250 trials, developed using EPrime 1.2 (Psychology Software Tools, Pittsburgh, PA, USA). During the task, participants were asked fixate on a cross visible on the screen. After 500 ms, the fixation cross disappeared and a white arrow was presented on screen for a total of 1,000 ms. Participants were asked to indicate the direction of the arrow by responding with their corresponding thumb as fast as possible. There were 93 Go-left trials and 95 Go-right trials. During the remaining 62 trials, a stop-signal (a large white cross) was superimposed over the arrow, requiring the participant to refrain from responding to the arrow. The stop-signal appeared after the so-called stop-signal delay (SSD). The SSD was set to 250 ms at the start of the experiment and updated by a tracking algorithm that used the participant’s performance on the task. When the participant successfully refrained from responding after de stop-signal, 50 ms of the SSD were subtracted. This caused the stop-signal to superimpose the arrow earlier. And after every unsuccessful stop-signal, 50 ms were added. This way, the success-to-error rate was around 50%. The Stop- and Go-trials were pseudorandomized; the first 12 trials were always Go-trials, and the Stop-trials never occurred consecutively. The inter-trial-interval differed throughout the task between 1,500-2,500 ms. The duration of the complete SST was around sixteen minutes.

*1.2 MRI acquisition*

The MRI scans were done at the Amsterdam UMC, location VU medical center, utilizing a Discovery MR750 3.0T MR scanner, using a 64-channel head coil. The MR images were acquired with 42 ascending slices per volume (slice thickness=3mm, inter-slice gap=0.33 mm, and 3.3x3.3 mm in-plane resolution) and a gradient echo-planar image sequence (TR=2.2 seconds, TS=26 ms, 64x64 matrix, field of view=21.1 cm, flip angle=90º). The head of the participant was immobilized during scanning, to minimize movement artifacts. Participant looked at the screen placed behind the MR scanner, where the tasks were visualized, through a mirror placed on the head coil. Responses of the participants were collected using an MR compatible response box (Current Designs, Philadelphia, PA, USA).

*1.3 TMS-EEG acquisition*

In total, four brain areas were stimulated using one single and two paired pulse TMS paradigms; one paradigm with 2 ms interval (D2; the SICI paradigm) and one with 10 ms interval (D10; the ICF paradigm). In both the D2 and D10 paradigm, the first stimulus was the condition pulse which was set at an intensity of 80% MT, and was followed by the test pulse at 120% MT. The single pulse paradigm contained one test pulse of 120% MT. Each stimulation paradigm consisted of 51 trials, resulting in a total of 153 trials per brain area. The order of the stimulation paradigms was randomized within one brain area (with an inter-trial-interval alternating between 4, 5 and 6 seconds), as well as the order of the brain areas to stimulate per participant. The localization of the brain areas differed; the left DLPFC and left preSMA were localized using the acquired brain areas by the TOL and SST brain activation, respectively. The right DLPFC was localized using the Beam F3/F4 method, and the left M1 localization was done during the MT determination.

*1.4 Preprocessing of (f)MR images*

The task-based fMR images were processed using SPM12 (Wellcome Trust Centre for Neuroimaging London). Functional images were manually reoriented to the structural T1 scan. The first three volumes were discarded and slice timing correction, scanulling (of >2mm of frame to frame displacement), normalization and spatial smoothing (using an 8 mm Gaussian kernel) was carried out during preprocessing. A high-pass filter (128-second cutoff period) was applied to remove low-frequency noise.

For the left DLPFC localization, the local maximum in its area (defined as Brodmann area 9 and 46) during the planning contrast retrieved from the TOL task (all planning conditions > baseline) was defined. If the peak voxel was not on top of a gyrus, the stimulation target was manually placed more on top to the closest gyrus. This was done in the participants T1 scan. For the left preSMA localization, we used the local maximum of the medial gyrus of Brodmann area 6 (anterior to the anterior commissure) during the response inhibition contrast (successful stop trials > successful go trials) of the SST. If no suitable local maximum could be detected, we used the following literature coordinates (in MNI space): Left DLPFC: -40, 28, 30 (7) and for left preSMA: -4, 14, 58 (8). For the right DLPFC, we retrospective determined the stimulation location on the T1 by extracting the entry and target location from the neuronavigation tool. Once the individualized stimulation coordinate was defined, a 5mm ROI was created, warped from MNI to subject space and overlaid on a T1 MRI scan of the individual participant, allowing navigation to the individualized stimulation location of the left DLPFC and preSMA during the session.

*1.5 EEG preprocessing steps*

TMS-EEG data was pre-processed using a two-step Independent Component Analysis (ICA) procedure in EEGLAB according to the TESA pipeline(Rogasch et al., 2017), running in MatLab 2022b. During the first step of preprocessing, automated removal of bad electrodes was performed in two stages. First, electrodes were removed based on the FlatlineCriterion (SD>5) and the LineNoiseCriterion (SD>4). Second, electrodes were removed based on kurtosis (SD>4). Afterward, epoch segmentation took place (1500ms before and after the TMS pulse), which was followed by a baseline correction using a time frame of 800ms until 110ms before the TMS pulse administration. During the second step of the preprocessing, large TMS pulse and TMS-evoked muscle artifacts were removed by removing 5ms before and 15ms after the TMS pulse administration. This was replaced utilizing cubic interpolation after which the data was down-sampled to 1kHz. Next, automated removal of bad epochs based on joint probability (SD>3) was performed, followed by a visual inspection of the epochs. For details, see (Rogasch et al., 2017).


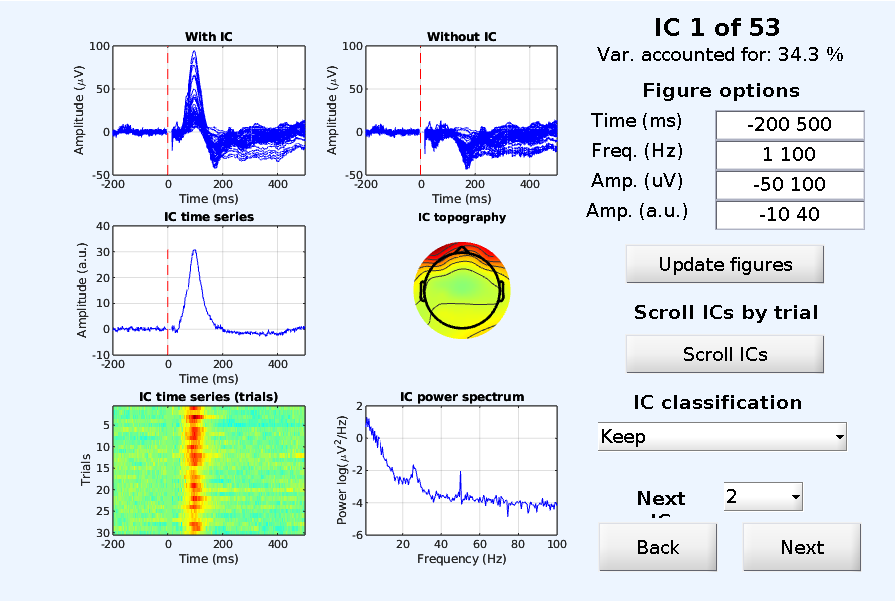

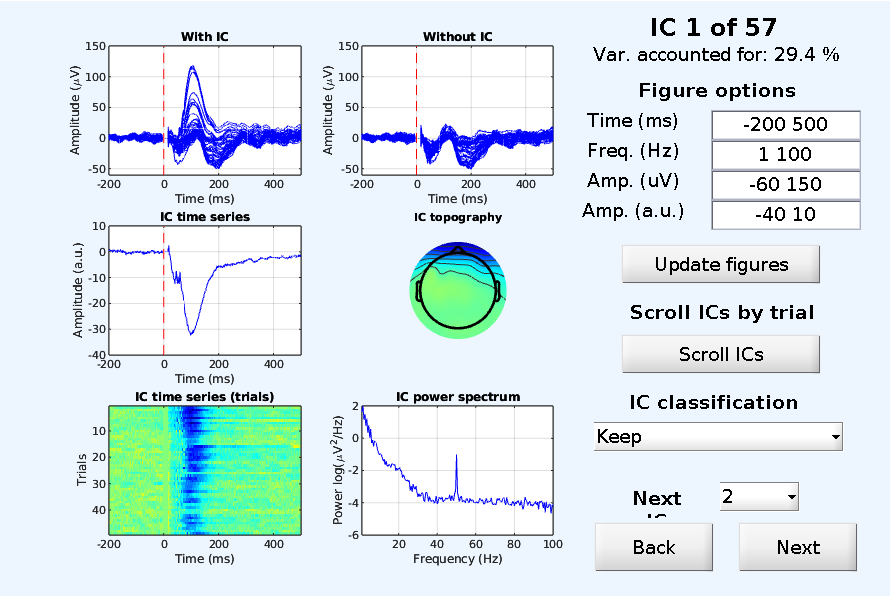

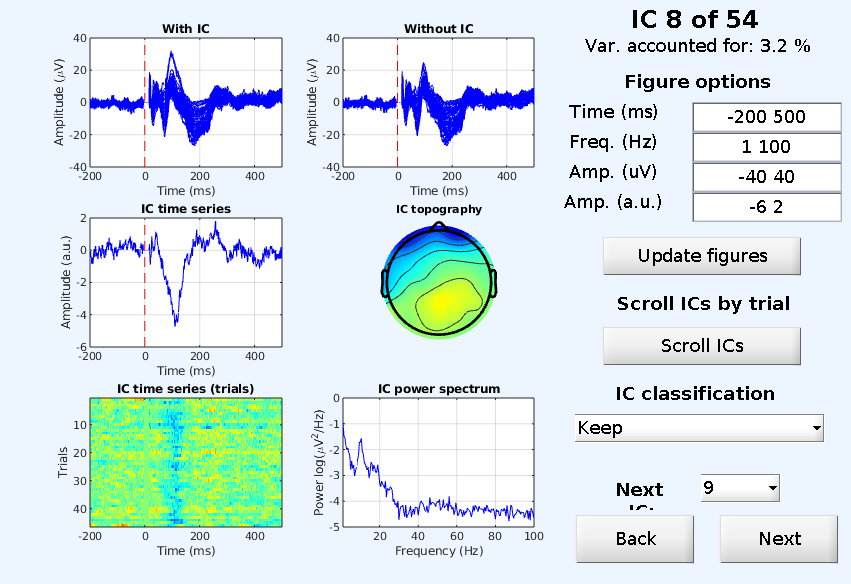

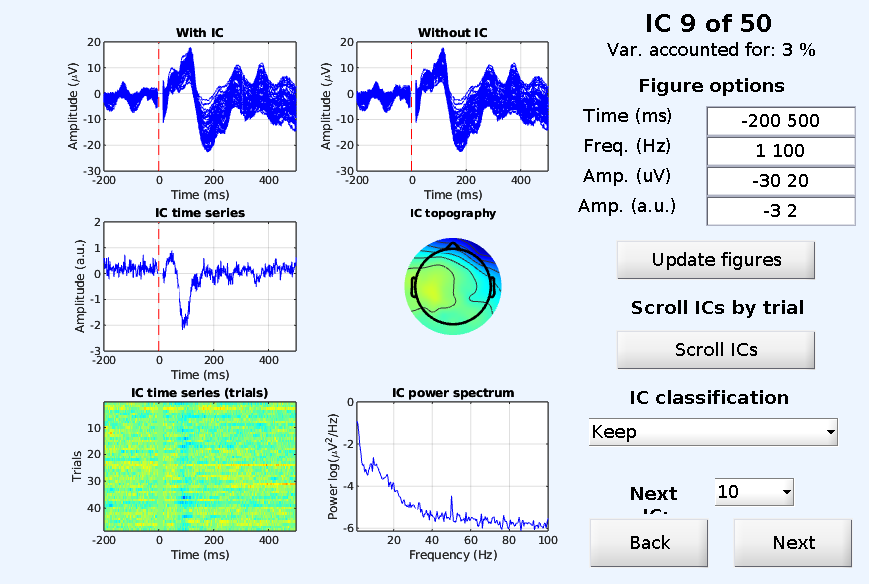
2. Results


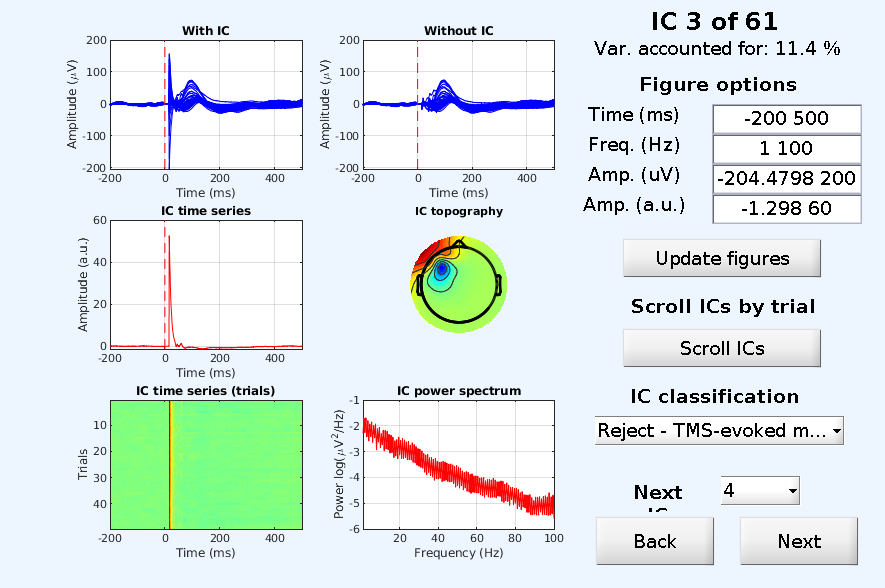

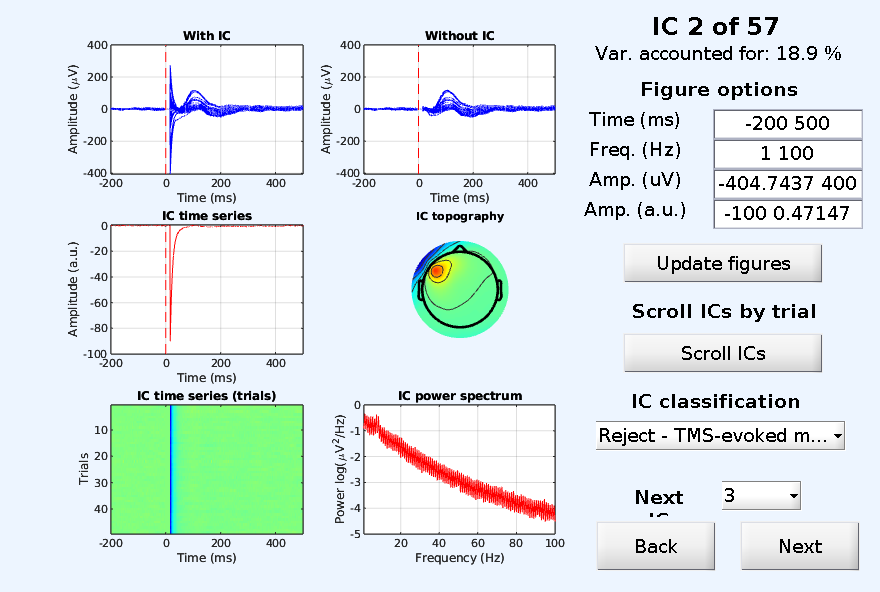


**Figure S2. Examples of TMS-evoked artifact during first ICA step (before filtering), after DLPFC stimulation**

**Figure S1. Examples of eye blinks during first ICA step (before filtering), after M1 (left) and DLPFC stimulation (right)**


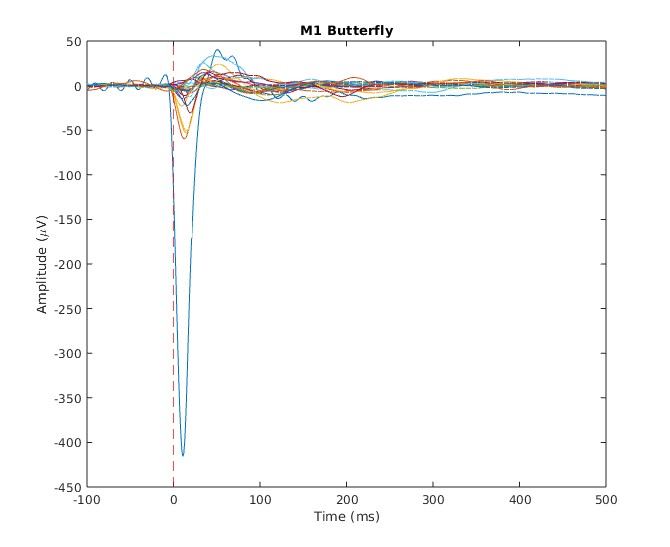

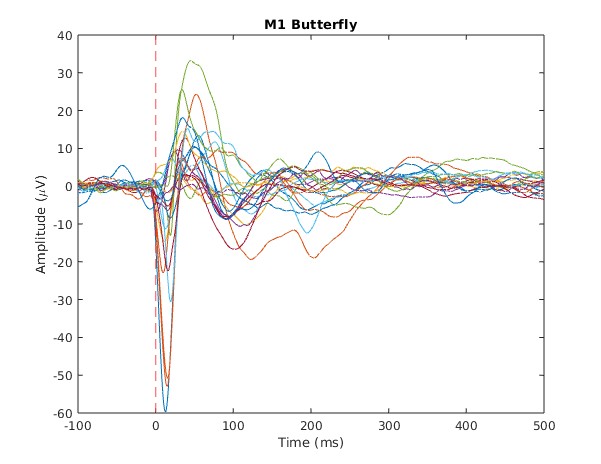

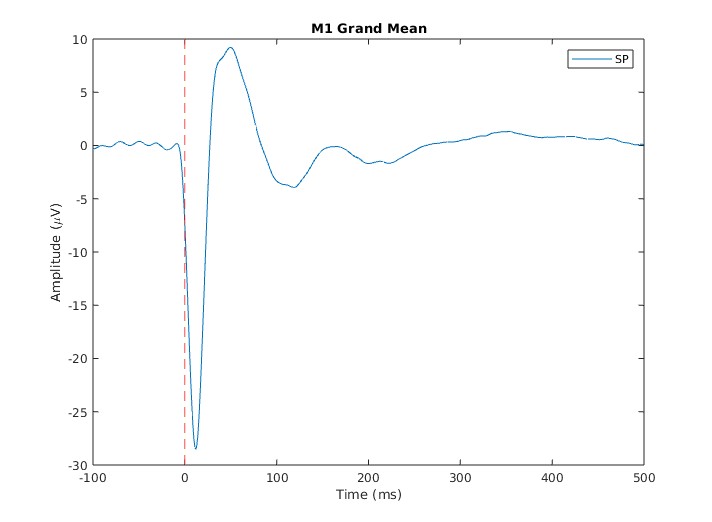

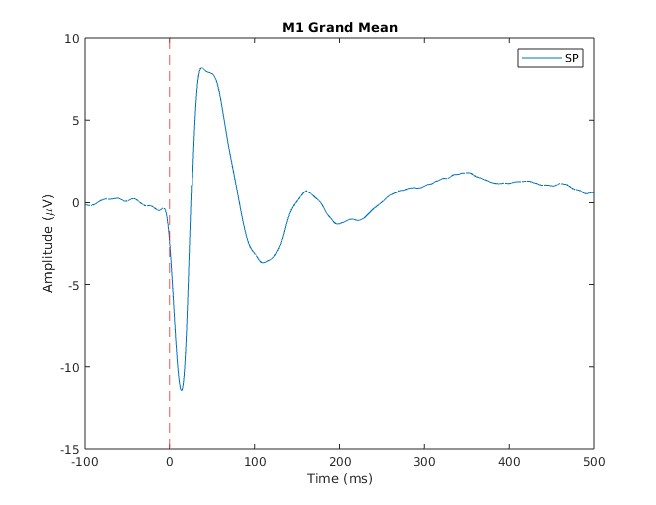


**Figure S3. Butterfly plots and grand mean after M1 stimulation with the outlier (top and bottom left) and without the outlier (top and bottom right).**


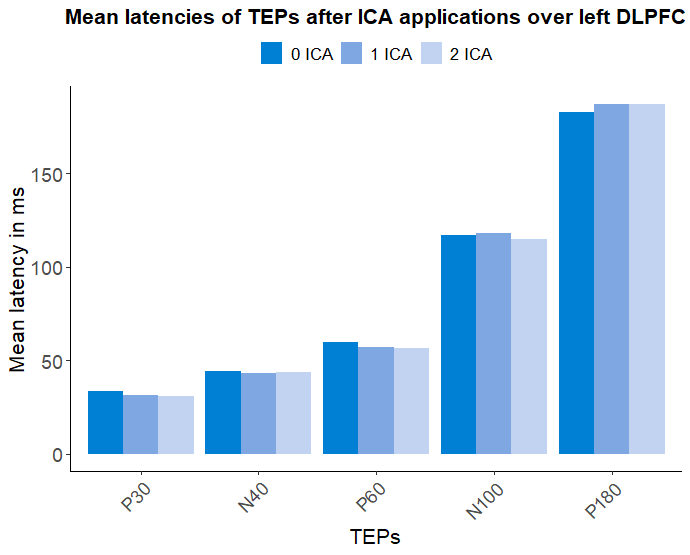

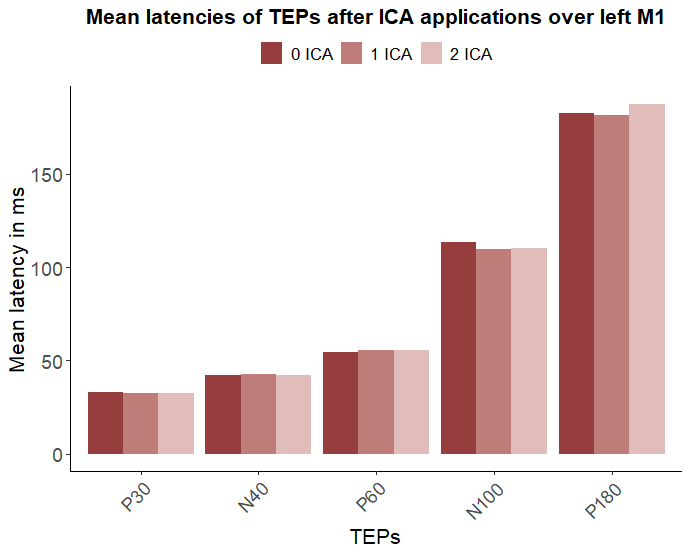


**Figure S4. Mean latencies of the individual TEPs after 0 (darker contrast), 1 (medium contrast) or 2 (light contrast) rounds of ICA after stimulation over the left DLPFC (in blue, left) and M1 (in red, right).**

**Figure S5. Butterfly plots and grand mean after left DLPFC stimulation with a bandpass of 1-48 Hz (top left and right), and normal filters (bandpass 1-80 Hz and bandstop 48-52 Hz).**

**
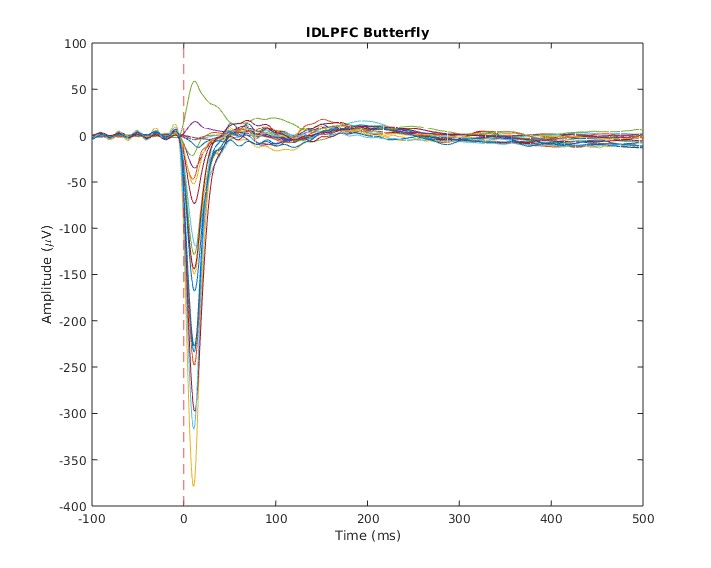

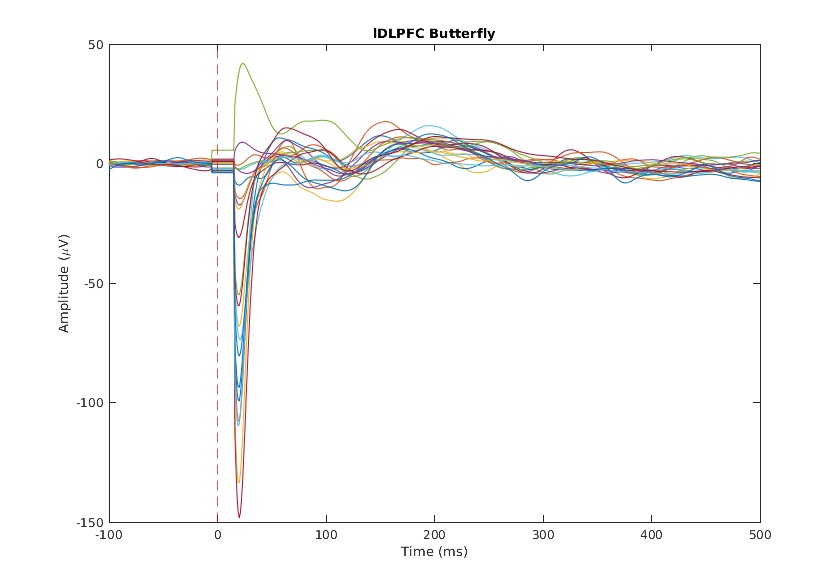

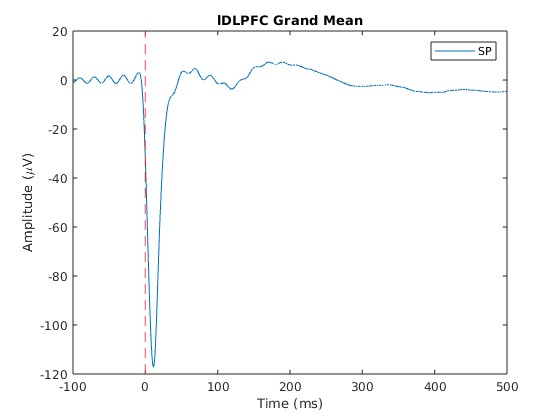

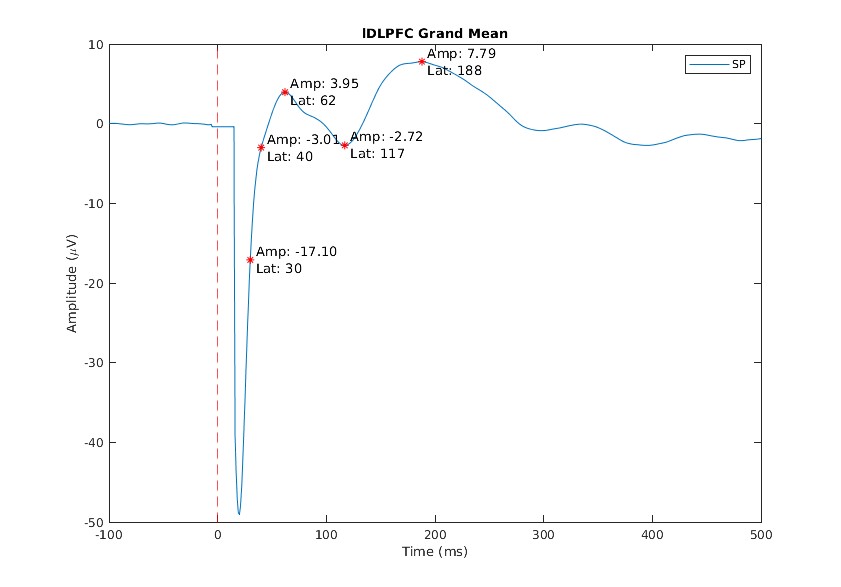
**


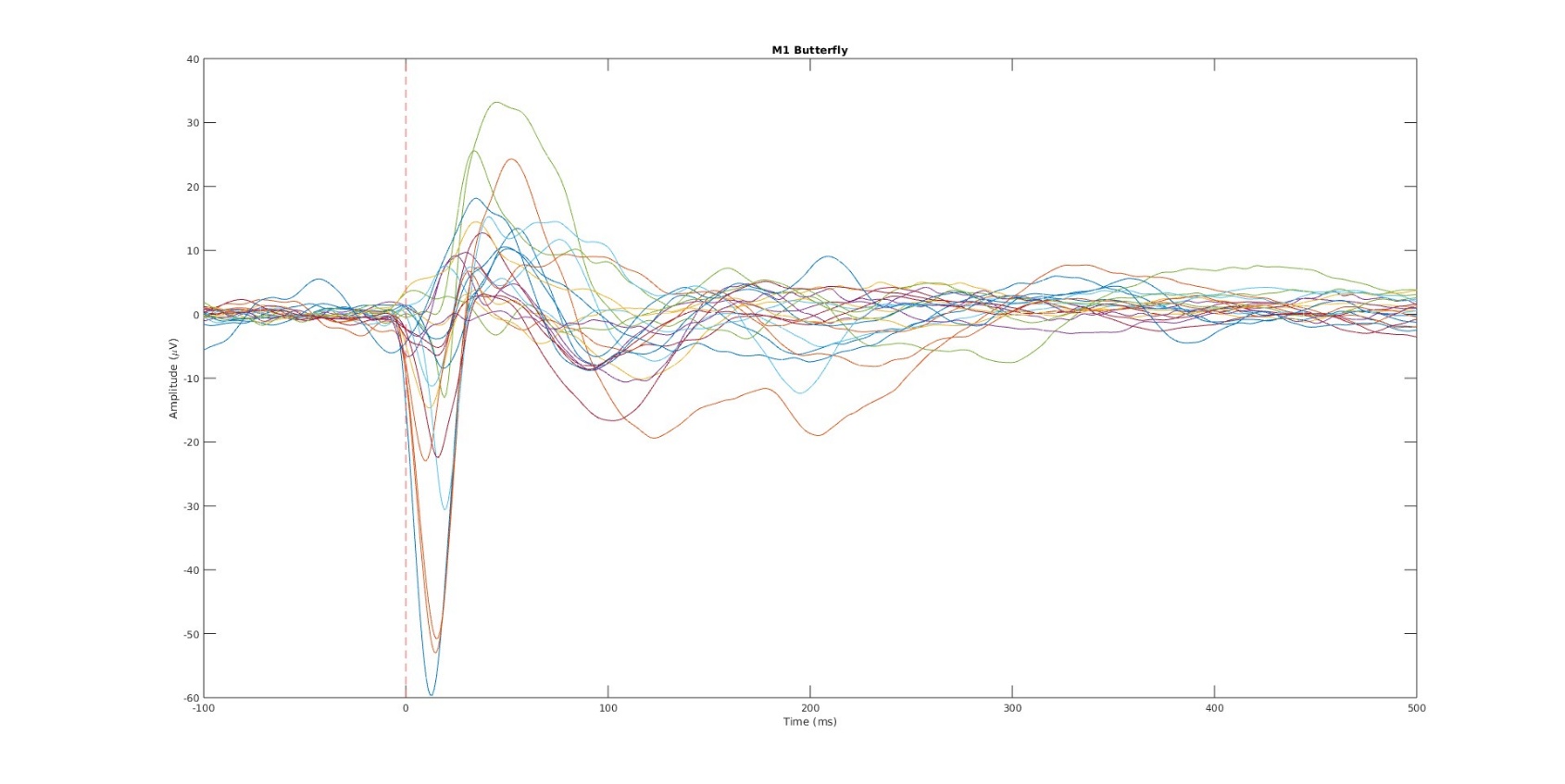

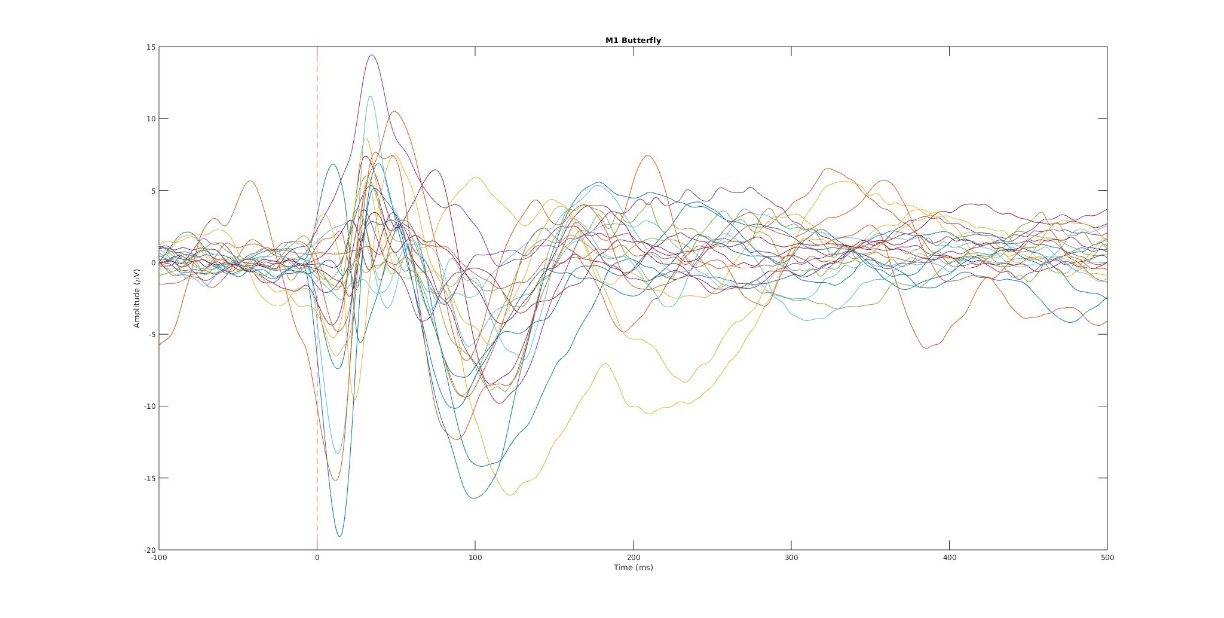

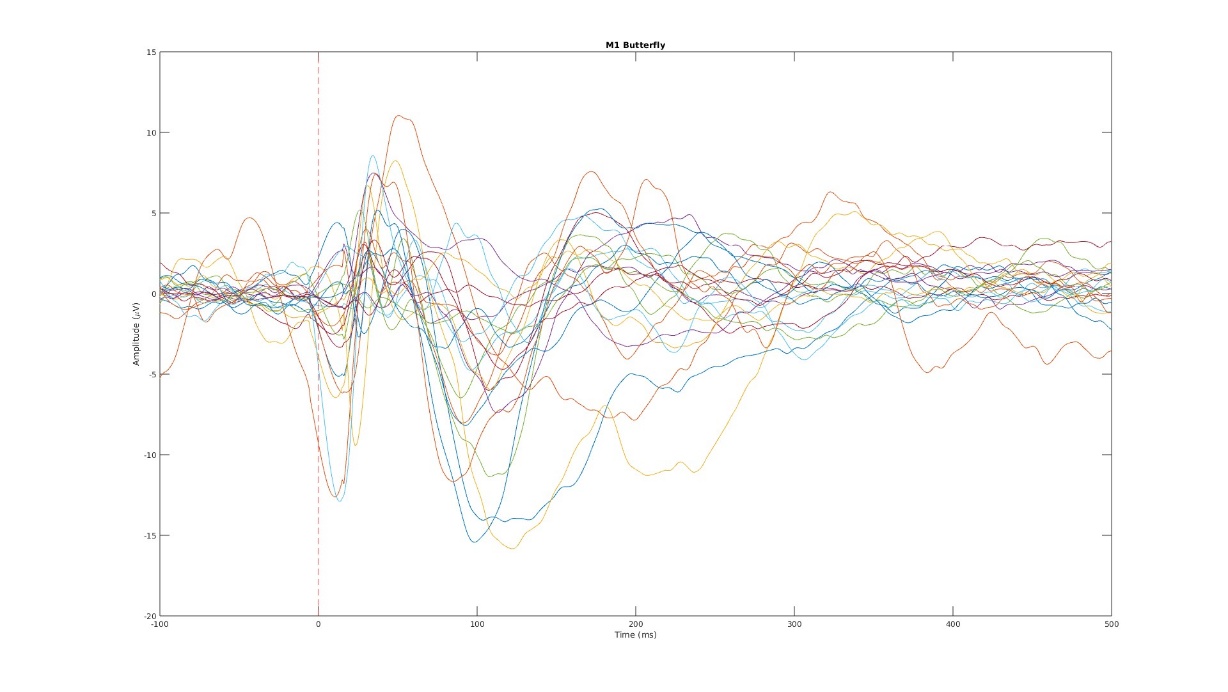


**Figure S6. Butterfly plots after M1 stimulation, using (top) zero, (middle) 1 or (bottom) 2 ICA rounds during preprocessing.**


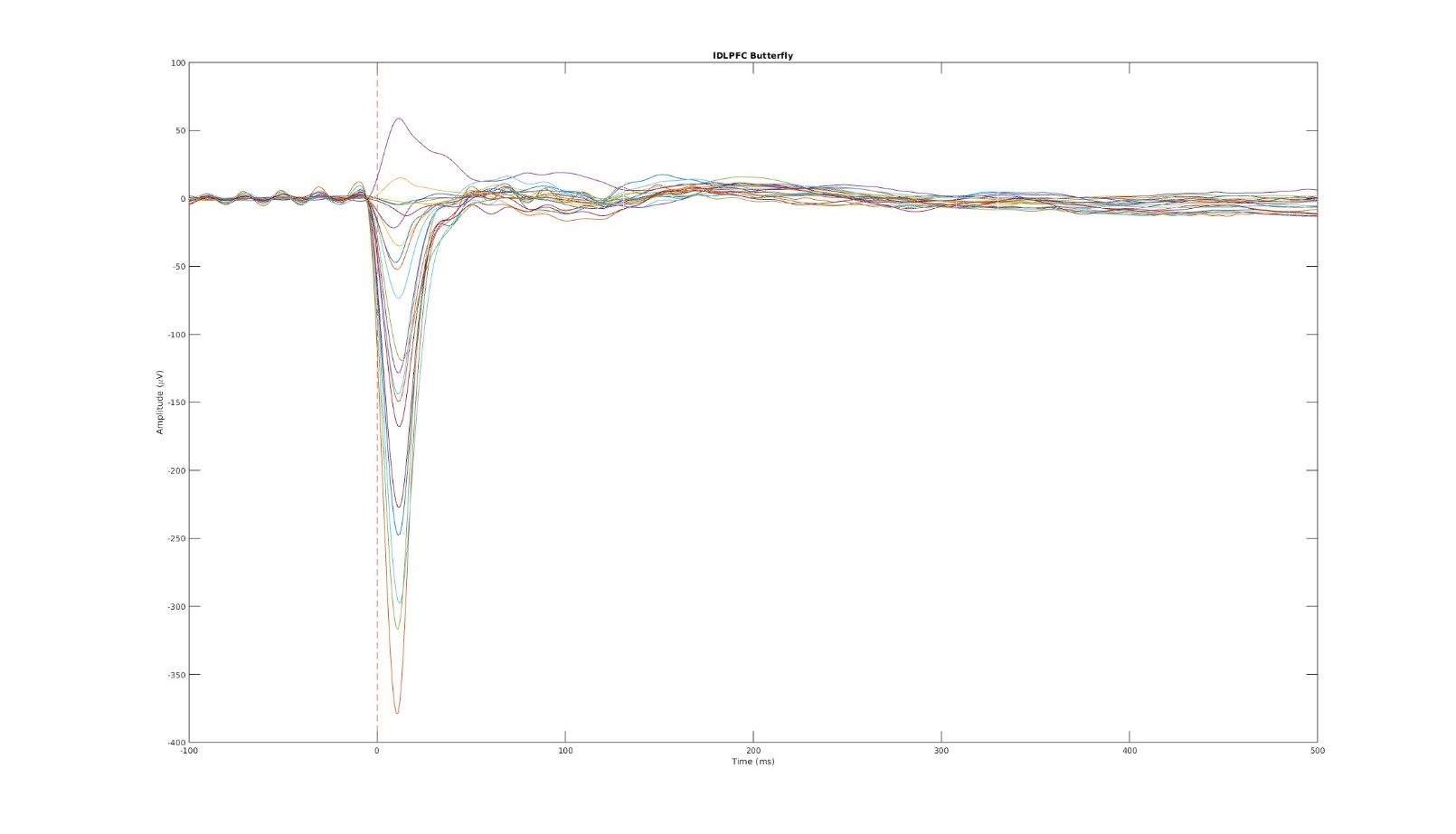

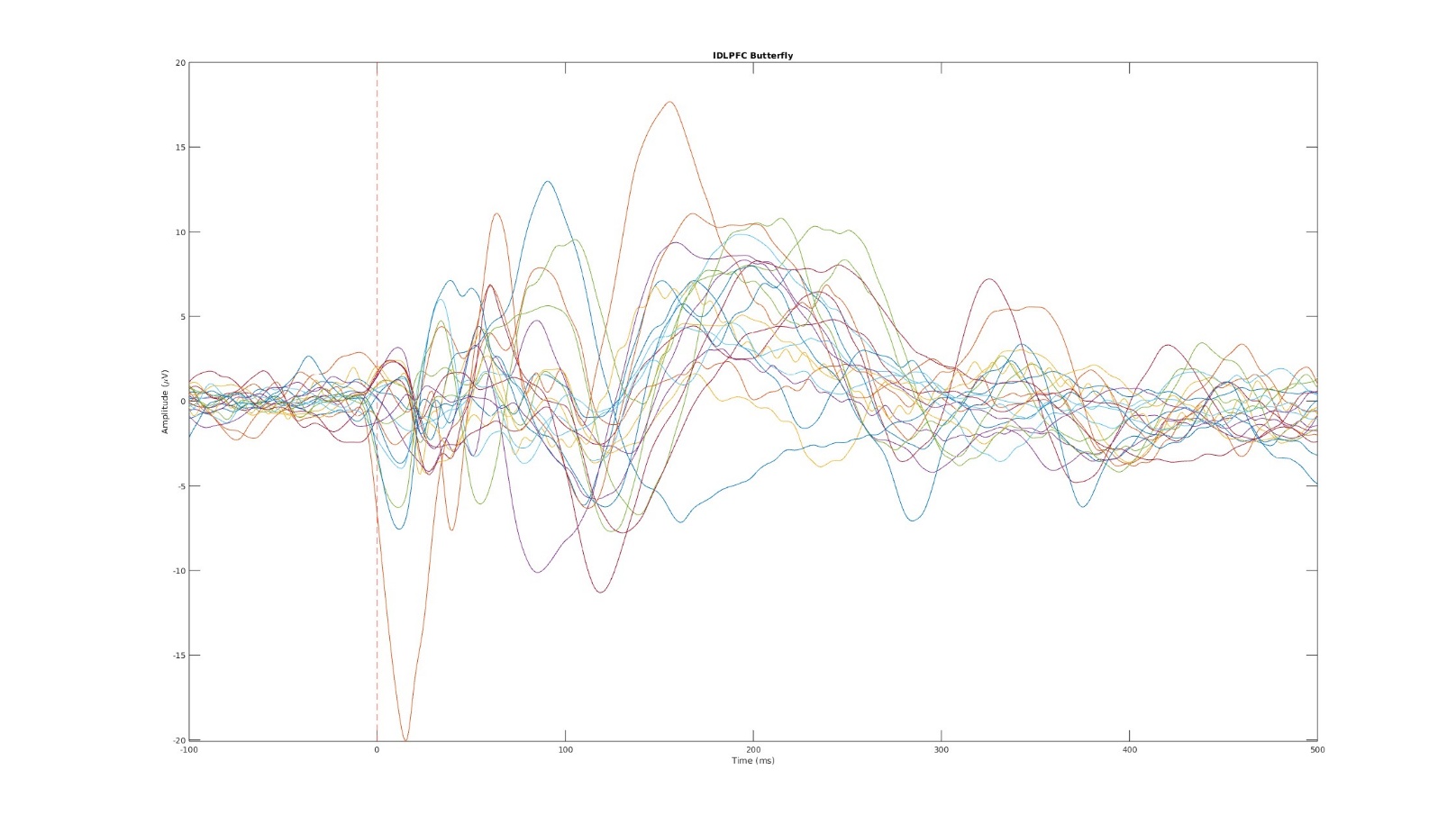

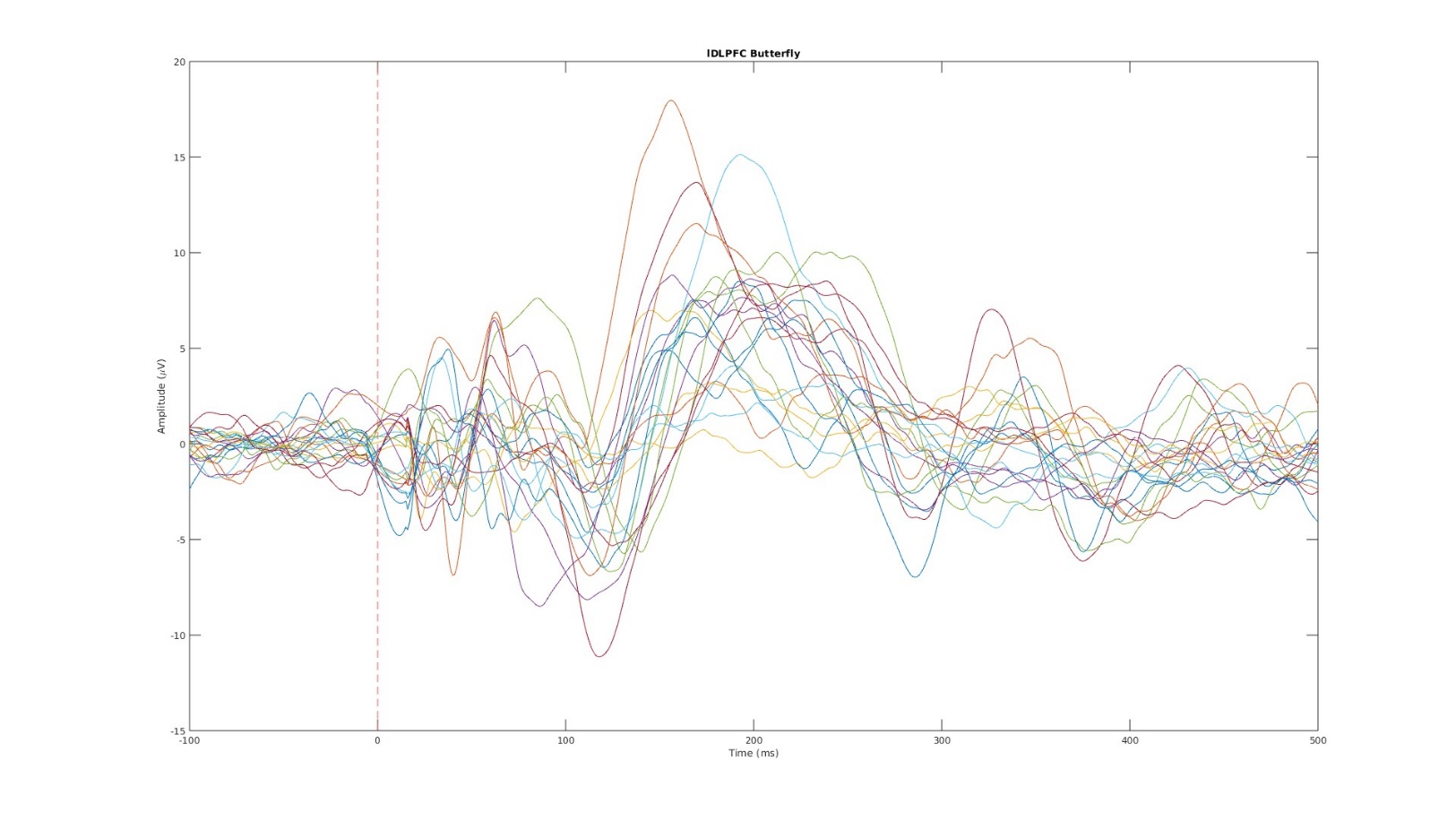


**Figure S7. Butterfly plots after lDLPFC stimulation, using (top) zero, (middle) 1 or (bottom) 2 ICA rounds during preprocessing.**

**References**

Fitzsimmons S, Postma TS, van Campen AD, Vriend C, Batelaan NM, van Oppen P, et al. Transcranial Magnetic Stimulation-Induced Plasticity Improving Cognitive Control in Obsessive-Compulsive Disorder, Part I: Clinical and Neuroimaging Outcomes From a Randomized Trial. Biol Psychiatry 2025;97(7):678-87. <https://doi.org/10.1016/j.biopsych.2024.06.029>.

Rogasch NC, Sullivan C, Thomson RH, Rose NS, Bailey NW, Fitzgerald PB, et al. Analysing concurrent transcranial magnetic stimulation and electroencephalographic data: A review and introduction to the open-source TESA software. Neuroimage 2017;147:934-51.
